# Gas bubble formation in the cytoplasm of a fermenting yeast

**DOI:** 10.1111/j.1567-1364.12004.x

**Published:** 2012-10-01

**Authors:** Chantel W Swart, Khumisho Dithebe, Carolina H Pohl, Hendrik C Swart, Elizabeth Coetsee, Pieter WJ van Wyk, Jannie C Swarts, Elizabeth J Lodolo, Johan LF Kock

**Affiliations:** 1 Department of Microbial, Biochemical and Food Biotechnology, University of the Free StateBloemfontein South Africa; 2 Department of Physics, University of the Free StateBloemfontein, South Africa; 3 Centre for Microscopy, University of the Free StateBloemfontein, South Africa; 4 Department of Chemistry, University of the Free StateBloemfontein, South Africa; 5 SABLtd Brewing Centre of ExcellenceAlrode, South Africa

**Keywords:** carbon dioxide, cell inclusions, fermentation, intracellular gas bubbles, NanoSAM, yeast

## Abstract

Current paradigms assume that gas bubbles cannot be formed within yeasts although these workhorses of the baking and brewing industries vigorously produce and release CO_2_ gas. We show that yeasts produce gas bubbles that fill a significant part of the cell. The missing link between intracellular CO_2_ production by glycolysis and eventual CO_2_ release from cells has therefore been resolved. Yeasts may serve as model to study CO_2_ behavior under pressurized conditions that may impact on fermentation biotechnology.

The fermentation process is at the heart of some of the most important biotechnological processes. This is demonstrated by the production of breads and alcoholic beverages where the fermenting capabilities of yeasts are exploited to produce ethanol and CO_2_. These conditions lead to cells capable of increased ethanol and CO_2_ production (Van Maris *et al*., 2001).

Although the yeast fermentation process is well established, no sign of CO_2_ bubbles inside cells has been reported. The lack of efforts to search for CO_2_ bubbles inside cells probably stems from the extensive work of Hemmingsen *et al*. (1990), whose research suggested that gas bubbles do not form within most types of cells even during gas supersaturation. This is ascribed to cytoplasmic resistance to gas bubble formation because of the increased structuring of water. Not even protein-enveloped gas vesicles that provide buoyancy to the cyanobacteria (Walsby, 1994) have been reported within yeast cells.

We used Nano scanning auger microscopy (NanoSAM) in both the scanning auger microscopy (SAM) and scanning electron microscopy (SEM) modes coupled with Argon-etching to show the gas bubble status inside individual brewer's yeast cells grown in fermentable and nonfermentable media, respectively.

The brewer's yeast, *Saccharomyces pastorianus* WS 34-70 (preserved at Cara Technology Limited, Leatherhead Enterprise Centre, Leatherhead, Surrey, UK), and the baker's yeast, *Saccharomyces cerevisiae* CBS 1171 NT (preserved at the Centraalbureau voor Schimmelcultures, Utrecht, the Netherlands), were cultivated in 500-mL conical flasks containing 100 mL media. Cells were grown to stationary phase at 25 °C while shaking at 160 r.p.m. in fermentable and nonfermentable media (Sherman, 2002). Fermentable media used was Glucose Yeast Malt broth (10 g L^−1^ glucose, 3 g L^−1^ yeast extract, 3 g L^−1^ malt extract, and 5 g L^−1^ peptone). The nonfermentable medium used, favoring respiration, was Yeast Peptone Glycerol broth (30 mL L^−1^ glycerol, 10 g L^−1^ yeast extract, and 20 g L^−1^ peptone). Samples were drawn from cultures in growth and stationary phases and subjected to Light Microscopy (Axioplan, Zeiss, Germany) to test purity and observe granular appearance. Cells were harvested by centrifugation at 1450 ***g*** for 5 min, washed with equal volumes of de-ionized water, and prepared for SEM, NanoSAM, and transmission electron microscopy (TEM) according to the study by Swart *et al*. (2010) and Kock *et al*. (2011). Experiments were performed in at least triplicate, and reproducible results were obtained. The brewer's yeast was analyzed by Light Microscopy, SEM, NanoSAM, and TEM, while the baker's yeast was analyzed by Light Microscopy and TEM as described. In addition, a *Microcystis aeruginosa* culture was subjected to TEM analysis as described by Swart *et al*. (2010).

To trace CO_2_ bubbles inside cells, the metal salt ZnSO_4_.7H_2_O (Merck, Germany) was added to the culture media, containing brewer's yeasts in fermenting mode, to a final concentration of 2 mg L^−1^ where after cells were prepared as aforementioned for NanoSAM analysis.

With this unique new application of nanotechnology, we could demonstrate the presence of a maze of coalescing bubbles that filled a significant part of fermenting cells (Fig. 1a; Supporting Information, Movie S1). A significant increase in the number and size of cross-sectioned bubbles were observed on surfaces of fermenting yeasts after Argon-etching (Fig. 1a and b; Table S1).

**Figure 1:**
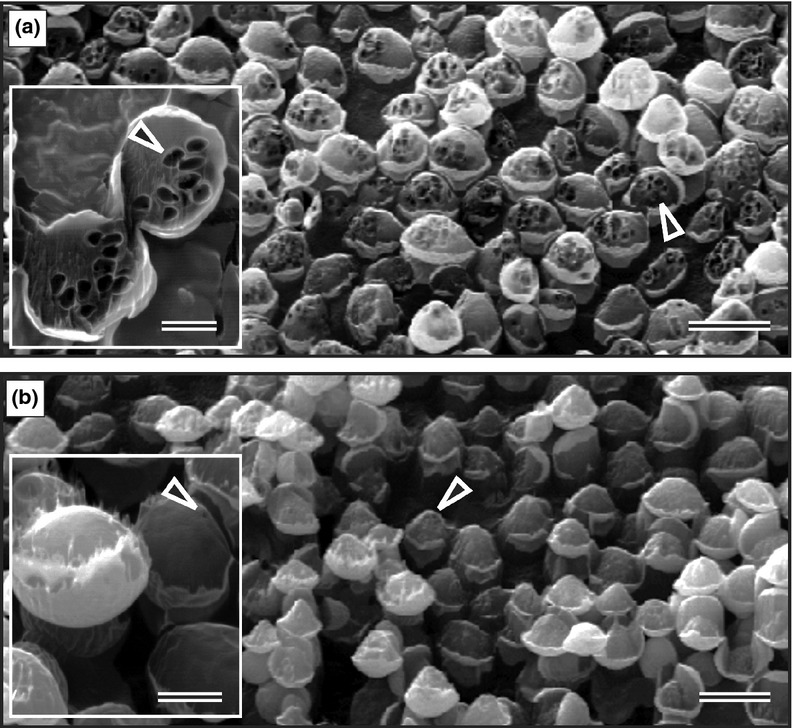
Bubbles (▸) within brewer's yeasts observed by NanoSAM Argon-etched SEM. (a) Fermenting cells with increased bubble formation. Insert: Cross-sectioned bubbles on etched surface. (b) Respiring yeasts with decreased bubble formation. Insert: Cross-sectioned bubbles on etched surface. Scale bars: (a, b)**=** 6 μm; (a, b) inserts = 2 μm.

To verify these striking results by an independent and nonrelated technique, we used the same TEM method with which we visualized protein-coated gas vesicles in a cyanobacterium (Fig. S1a), to show the naked cross-sectioned bubbles in brewer's yeast (Fig. S1b–d). The TEM-observed bubbles were of similar size to the bubbles observed in NanoSAM-SEM Argon-etched cells (Fig. a and b; Table S1). A similar trend in bubble formation was found in the model baker's yeast, *S. cerevisiae*, when grown in fermentable and nonfermentable media (Table S1). We could identify bubbles as granules in both the baker's and brewer's yeasts using Light Microscopy directly on living cells (Fig. S1e and f), thereby verifying both the NanoSAM- and TEM-demonstrated bubble formation in cells. This is reminiscent of gas vesicles observed by Light Microscopy in the cyanobacteria (Walsby, 1994). These results imply a wide distribution of the CO_2_ bubble formation phenomenon in fermenting *Saccharomyces* species. We found increased bubble production in young as well as older fermenting cells, suggesting that bubble production and fermentation were not strictly linked to cell age (Movie S1).

As expected, we observed ‘galvanized’ bubbles inside brewer's yeasts after the addition of zinc in the form of ZnSO_4_.7H_2_O to the growth medium. This suggested that zinc reacted with carbonic acid to form insoluble or weakly soluble metal bicarbonate at neutral pH in the cytoplasm (Ryan & Bauman, 1978). Carbonic acid should be produced at higher concentrations nearer to the boundary of the bubble if CO_2_ were present inside it, because of the reaction CO_2_ + H_2_O ⇌ H_2_CO_3_ in the cytoplasm (Fig. S1g and h). This mechanism may sequester metals to protect cells against toxicity when an excess of metal is present (Eide, 2006).

Based on the above and the fact that the observed empty bubbles were not collapsed by reported high intracellular osmotic pressure that may reach 2.1 MPa (Vella *et al*., 2012), we conclude that these intracellular bubble-like holes are gas bubbles containing CO_2_. We suggest that intracellular CO_2_ may eventually be secreted by pressure through the yeast cell wall to affect pressure homeostasis. This in turn should result in vigorous bubble release under diminished pressure (decompression) of the external environment, resulting in their coalescence and enlargement to visible bubbles of millimeter and centimeter size as is generally experienced in products of fermentation such as leavened bread, traditional beer, and champagne. Internal cell pressure is probably needed in these yeasts to keep bubble size at a minimum to decrease any adverse effects on cell function. Alternatively, these bubbles may be in nonpressurized transit as they are shipped out of the cell upon production.

An important question that needs to be answered is what stabilizes the bubbles in the cell? According to the study by Blasco *et al*. (2011), there are several cellular components of yeasts that are involved in foam formation and stabilization in fermented beverages. These surface active compounds may also be responsible for bubble stabilization inside the cell. Additionally, the potential meliorating effects of zinc and other inorganic ions on intracellular CO_2_ gas bubble structures warrant further investigation. The effects, control, and kinetics of intracellular gas bubbles at molecular and cellular levels as well as their impact on biotechnological processes should now be assessed. More generally, the yeast-bubble phenomenon may serve as a model that will provide a better understanding of the origins and effects of CO_2_ in biology, food, medicine, physics as well as the environment. We expect that this work will advance research on gas exchange in prokaryotic and eukaryotic cells and, for example, diving mammals where gas bubbles are formed in tissues under elevated pressurized conditions (Hooker *et al*., 2012).

## Statement

Re-use of this article is permitted in accordance with the Terms and Conditions set out athttp://wileyonlinelibrary.com/onlineopen#OnlineOpen_Terms

## Supporting Information

Fig. S1. Bubble (▸) and gas vesicle (→) analyses in the brewer's yeast and cyanobacterium, *Microcystis*, respectively.

Movie S1. NanoSAM-SEM video showing sequential Argon-etching through a dense maze of gas bubbles inside fermenting brewer's yeast cells of different ages that is older mother cell (center) with attached younger daughter cells.

Table S1. Cell size (diameter) and gas bubble (Bub) number and diameter dimensions inside brewer's and baker's yeasts grown in fermentable and non-fermentable media.
