# Supplementary figures and images for: Gas bubble formation in the cytoplasm of a fermenting yeast
Source: FEMS Yeast Res. 2012 Oct 1;12(7):867–9. doi: 10.1111/j.1567-1364.12004.x (PMC3503256; doi:10.1111/j.1567-1364.12004.x)

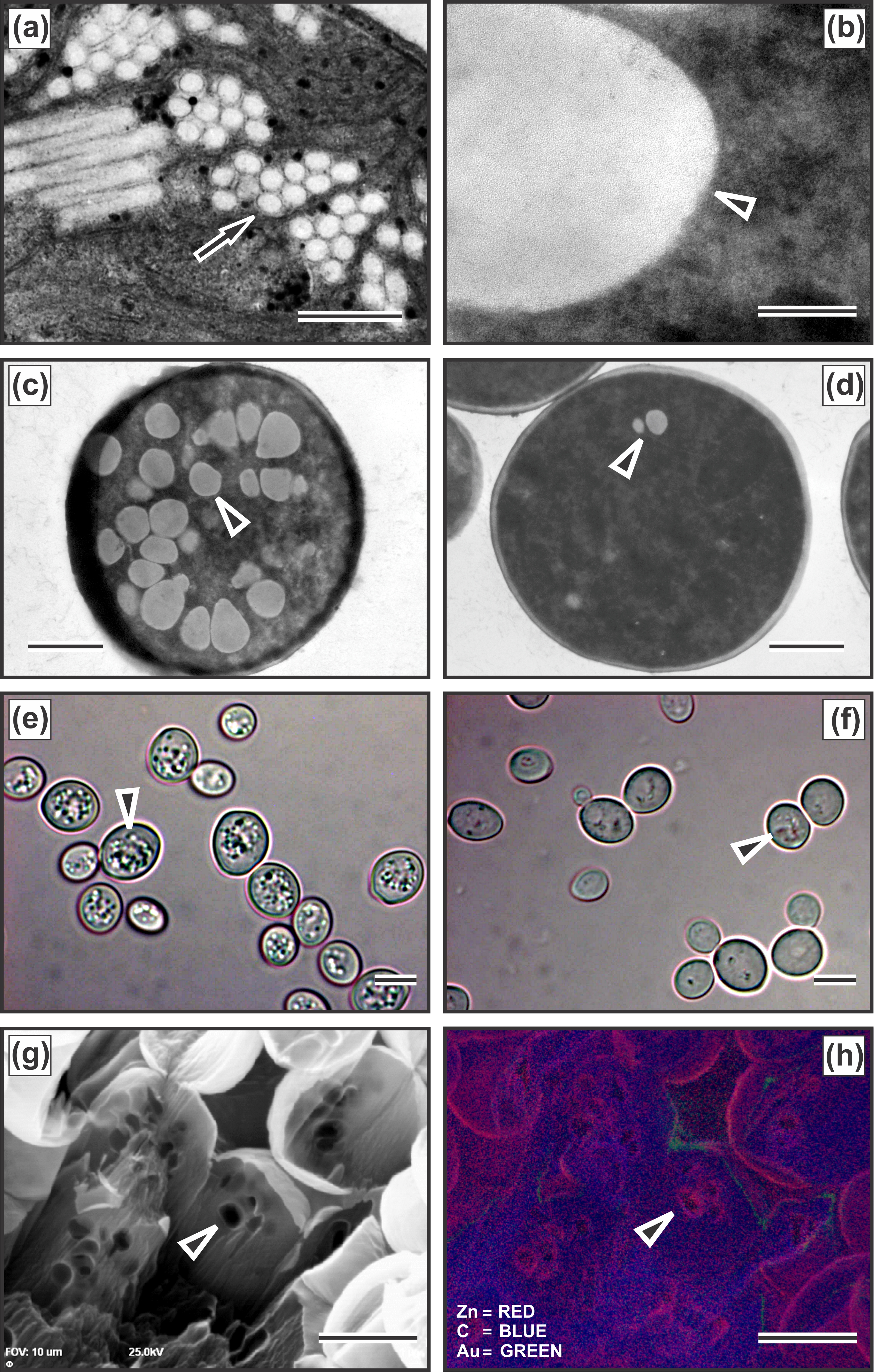

Supplement: Fig. S1. — Bubble (▸) and gas vesicle (→) analyses in the brewer's yeast and cyanobacterium, Microcystis, respectively. [file fyr0012-0867-FigS1.jpg]
