# Supplementary material for: Gas bubble formation in the cytoplasm of a fermenting yeast
Source: FEMS Yeast Res. 2012 Oct 1;12(7):867–9. doi: 10.1111/j.1567-1364.12004.x (PMC3503256; doi:10.1111/j.1567-1364.12004.x)
Supplement: Table S1. — Cell size (diameter) and gas bubble (Bub) number and diameter dimensions inside brewer's and baker's yeasts grown in fermentable and non-fermentable media. [file fyr0012-0867-TableS1.docx]

**Table S1.** Cell size (diameter) and gas bubble (Bub) number and diameter dimensions inside brewer’s and baker’s yeasts grown in fermentable and non-fermentable media

______________________________________________________________________________

Fermentable media Non-fermentable media

**Brewer’s yeast**

Bub number 4.8 ± 2.7 0.8 ± 1.4

Bub Size (nm) 547 ± 228 392 ± 139

Cell size (µm) (3.6 – 4.8) x (3.7 – 5.3)

**Baker’s yeast**

Bub number 7.8 ± 4.4 1.0 ± 1.3

Bub size (nm) 564 ± 116 278 ± 120

Cell size (µm) (3.2 - 4.8) x (3.6 - 6.1)

______________________________________________________________________________

^The table shows a significant increase in the number (P < 0.0001, n = 50; Two tailed t-Test) and size (P < 0.05, n = 50; Two tailed t-Test) of bubbles in the brewer’s and baker’s yeasts respectively when cultivated in fermentable medium [increased CO^_2_ ^production] compared to when grown in a non-fermentable medium (decreased CO^_2_ ^production). A similar number and size of bubbles inside brewer’s yeasts when grown in fermentative and non-fermentative media respectively, were observed with Nano Scanning Auger Microscopy (NanoSAM) linked to Scanning Electron Microscopy (SEM) and Argon etching as well as when only Transmission Electron Microscopy (TEM) was used. Results for baker’s yeasts were obtained by TEM. The dimensions of 50 yeast cells grown in fermentable medium were determined by Light Microscopy.^
